# Relationship of family formation characteristics with unsafe abortion: is it confounded by women’s socio-economic status? - A case–control study from Sri Lanka

**DOI:** 10.1186/s12978-016-0173-5

**Published:** 2016-06-17

**Authors:** Carukshi Arambepola, Lalini C. Rajapaksa, Deepika Attygalle, Loshan Moonasinghe

**Affiliations:** Department of Community Medicine, Faculty of Medicine, University of Colombo, Kynsey Road, Colombo 8, Sri Lanka; University of Colombo, Kynsey Road, Colombo 8, Sri Lanka; Family Health Bureau, Ministry of Health, de Saram Place, Colombo 8, Sri Lanka

**Keywords:** Family formation characteristics, Unsafe abortion, Socio-economic status

## Abstract

**Background:**

Literature shows that choice for unsafe abortion is often driven by poverty. However, factors related to the family formation behaviour of women are also implied as determinants of this decision. This study assessed which family formation characteristics of women are associated with the risk of unsafe abortion, without being confounded by their low socio-economic status among Sri Lankan women admitted to hospital following post-abortion complications.

**Methods:**

An unmatched case–control study was conducted in nine hospitals in eight districts in Sri Lanka among 171 women with post-abortion complications following unsafe abortion (Cases) and 600 postpartum mothers admitted to same hospitals during the same period for delivery of term unintended pregnancies (Controls). Interviewer-administered-questionnaires obtained demographic, socio-economic and family formation related characteristics. Risk factors of abortion were assessed by odds-ratio (OR), adjusted for their socio-economic status in logistic regression analysis.

**Results:**

Low socio-economic status, characterised by low-education (adjusted OR = 1.5; 95 % CI = 1.1–2.4) and less/unskilled occupations (2.3; 1.4–3.6) was a significant risk factor for unsafe abortion. Independent of this risk, being unmarried (9.3; 4.0–21.6), failure in informed decisions about desired family size (2.2; 1.4–3.5), not having a girl–child (2.2; 1.4–3.4) and longer average birth intervals (0.7 years; 0.6–0.8) signified the vulnerability of women for unsafe abortion. Cases were as fast as the controls in their family completion (4.3 versus 4.5 years; *p* = 0.4), but were at increased risk for abortion, if their average birth intervals (including the last one) were longer. Previous contraceptive use, age at reproductive events or partners’ characteristics did not impart any risk for abortion.

**Conclusions:**

Low socio-economic status is not the most influencing risk factor for unsafe abortions leading to complications, but many other factors in relation to their family formation characteristics that are independent of their low socio-economic status.

## Background

Induced abortion is termination of an unintended pregnancy through deliberate intervention. It is considered ‘unsafe’ when carried out either by persons lacking the necessary skills or in an environment lacking the minimum medical standards, or both [1]. More than 50 % of all induced abortions worldwide are unsafe, of which more than 98 % are performed in the low- and middle-income countries [2]. Approximately 47,000 die of complications following unsafe abortions each year, giving a case fatality rate of 220 deaths per 100,000 unsafe abortions. This rate is nearly 350 times higher than that associated with legal induced abortion [2, 3].

South Asia is home to nearly one third of the world’s population. Sri Lanka stands out in this region because of its impressive health indicators, which are comparable to those of high-income countries [4]. The national family health programme in Sri Lanka has received many accolades for reducing the maternal mortality from 2680 in 1936 to 32.5 per 100,000 live births by 2013 [5]. Its further reduction is however a challenge that requires interventions specifically targeting the women vulnerable to easily preventable causes of maternal death [6]. Septic abortion, occurring as a direct result of unsafe abortion has remained a significant contributor to both maternal morbidity and mortality in Sri Lanka, with no declining trend over the past few years [5, 7, 8].

Abortion is illegal in Sri Lanka unless performed as a measure to save a pregnant woman’s life [9, 10]. State health facilities which function under the ‘free for all’ health policy in the country do not provide abortion services. Yet, though illegal, safe abortion services are available elsewhere. However, some women have limited access to these services, thus resort to unsafe abortions and almost always succumb to post-abortion complications. Identifying the risk factors of unsafe abortion particularly of these women who may require treatment for complications is vital, since they are the most vulnerable for maternal morbidity out of all women who undergo induced abortion.

In low- and middle-income countries, the majority of women undergoing unsafe abortion are predominantly of poor education, and in less skilled or unskilled occupations, thus the choice of unsafe abortion is believed to be driven by low socio-economic status [11–14]. Nonetheless, studies from the same region have consistently shown that several other factors such as women’s marital status, decisions made with partners on desired family size, achievements in reproduction and completion of their families also play an important role as risk factors for unsafe abortion [15–20]. All these factors are implied in the formation of a woman’s ‘desired family size’ (hence called family formation characteristics), most of which are also associated in many ways with their low socio-economic status. As such, which family formation characteristics would actually contribute directly as risk factors for unsafe abortion without being confounded by their low socio-economic status, remains inconclusive. Merely descriptions of women undergoing unsafe abortion with no reference made to a comparison group or attempts to quantify the risk associated with family formation characteristics with no adjustments made for their low socio-economic status, fail to shed light on the link between these factors.

At individual level, family formation characteristics of women are modifiable through proper planning of the family. Identifying specific family formation characteristics that do associate with unsafe abortion, not confounded by their low socio-economic status, would provide a more pragmatic approach, especially in settings where unsafe abortion is more prevalent among the poor, so that even poorer women having such risk factors could be specifically targeted for early measures on optimal family formation. In this backdrop, the objective of our study was to assess the risk of unsafe abortion associated with family formation characteristics, not confounded by the socio-economic status of women who develop post-abortion complications requiring treatment. It is believed that the findings of this study would be applicable to similar settings in both high and low- and middle-income countries.

## Methods

We carried out an unmatched case–control study in nine state hospitals in eight out of the 25 districts in Sri Lanka over a period of six months. Five included were hospitals reporting the highest number of abortions for any given district, and the others purposively selected for adequate representation of the minority ethnic groups and the apex referral hospital for women in Sri Lanka.

Cases were women admitted to the selected hospitals for treatment of complications following an unsafe abortion. Controls were mothers admitted to postnatal wards in the same hospitals following the delivery of an unintended pregnancy carried to term. The required sample size was 159 cases and 600 controls. Potential cases were identified by screening the women presenting to gynaecology and medical/surgical casualty wards, and based on the WHO definitions [21], they were recruited under three categories: ‘certainly induced (based on woman’s statement and/or genital trauma or evidence of manipulation or foreign body in the genital tract), ‘probably induced’ (based on sepsis/peritonitis and unintended pregnancy) and ‘possibly induced’ (based on sepsis/peritonitis or unintended pregnancy). Definition on ‘unintended pregnancy’ [21], calculation of the sample sizes and how the cases and controls were recruited to the study are published in detail elsewhere [22, 23].

Data on women’s age, socio-economic status (education level, current employment status and type of occupation of women and partner) and family formation characteristics (marital status, fertility behaviour, ever-use of contraceptives, number of pregnancies, living children, duration of each birth interval) were collected using an interviewer-administered questionnaire by pre-intern medical officers who were not part of the team providing care. Details on the development of questionnaires are published elsewhere [21–23].

‘Low’ level of education was defined by not having completed secondary education leading to the General Certificate of Ordinary Level examination. ‘Less/un-skilled’ category of employment was defined by elementary occupations and occupations of plant and machine operators and assemblers, and ‘skilled’ category defined by all other occupations. Type of current employment of women was operationalised based on employment status and type of occupation. Fertility behaviour was operationalised based on informed decisions made with partners on desired family size and completion of family after making such decisions.

Prior to data collection, interviewers were trained by a group of psychologists and experts in qualitative research on obtaining sensitive data. Data collection was done after building good rapport with each participant and over several days in the privacy of a separate area in the ward to ensure quality data.

### Ethics, consent and permission

Ethics clearance for the study was granted by the Ethics Review Committee of the Faculty of Medicine, University of Colombo, Sri Lanka. Written informed consent was obtained from each participant. While ensuring anonymity and confidentiality, sensitive questions were administered only at the end of interview. Data were not revealed to the hospital staff or family members. Reading material on the contraceptive services available at field level was distributed.

### Data analysis

Data were analysed using Statistical Package for Social Sciences (SPSS) Version 20.

Initially, the risk of abortion associated with each characteristic related to the socio-economic status and family formation characteristics of the participants was assessed in uni-variate analysis, using odds ratio (OR) and 95 % confidence interval (CI) for categorical data and applying *t* test for quantitative data. Subsequently, logistic regression analysis was performed to assess the role of each factor in the risk of unsafe abortion, after controlling for the confounding effect of socio-economic status of women. In the regression model, all the factors that were significant in the uni-variate analysis were included as independent variables, while the dependent variable was the case–control status of women. The model assessed the risk using backward likelihood ratio method at 0.05 probability of exit at each step.

The relationship of unsafe abortion with the length of birth interval was further illustrated, by plotting the median age at every pregnancy of women in each group of gravida (1–6) against the order of pregnancies in a graph, separately for cases and controls. Mean age of cases and controls at major reproductive events were also illustrated in graph among women who had completed their families.

## Results

A total of 171 women were included as cases and 600 postpartum mothers as controls. To ensure that cases represented women who developed complications following unsafe abortion, of all the women recruited as potential cases, only the women who showed definitive clinical signs of infection and received intravenous antibiotic treatment in the ‘probably induced’ abortion category (*N* = 49) and all in the ‘certainly induced’ abortion category (*N* = 122) were considered as cases for final analysis.

The mean age of sample was 30.6 years (SD = 6.3) ranging from 15 to 46 years. The majority of women were of Sinhalese ethnicity (67.1 %), married at the time of unintended pregnancy (94.7 %), poor education (58.2 %) and unemployment status (71.7 %). Nearly 34 % were in their third pregnancy while most of the employed women were working as manual labourers or factory workers.

Tables 1 and 2 show the risk factors of unsafe abortion that led to complications among women who had an unintended pregnancy. All the family formation and socio-economy related risk factors that were identified in the uni-variate analysis remained significant, when assessed for their independent association with unsafe abortion in the logistic regression model. The model exhibited significance at 0.01 level with an 80.6 % overall percentage correct prediction.

**Table 1:** Risk factors of unsafe abortion in relation to socio-economic status among women with unintended pregnancies

Socio-economic status	Cases		Controls		Crude OR (95 % CI)	Adjusted OR (95 % CI)^b^
	*N* = 171^a^		*N* = 600^a^			
	No.	%	No.	%		
Secondary education						
Not completed	115	67.6 %	332	55.5 %	1.7 (1.2–2.4)	1.5 (1.1–2.4)
Completed (Reference)	55	32.4 %	266	44.5 %	1.00	1.00
Type of current employment						
Unskilled/Less skilled	52	30.6 %	80	13.5 %	2.9 (1.9–4.3)	2.3 (1.4–3.6)
Skilled	16	9.4 %	62	10.5 %	1.1 (0.6–2.0)	1.3 (0.7–2.4)
None (Reference)	102	60.0 %	451	76.0 %	1.00	1.00
Secondary education of partner						
Not completed	89	59.7 %	308	52.1 %	1.4 (0.9–1.9)	-
Completed (Reference)	60	40.3 %	283	47.9 %	1.00	
Partner currently employed^c^						
No	5	3.1 %	3	0.5 %	6.3 (1.5–26.3)	
Yes (Reference)	158	96.9 %	596	99.5 %	1.00	-
Type of occupation of partner^c,d^						
Unskilled/Less skilled	64	66.7 %	202	58.2 %	1.4 (0.9–2.3)	-
Skilled (Reference)	32	33.3 %	145	41.8 %	1.00	

^a^In some variables, row values do not add up to the total cases and controls due to missing data

^b^Adjusted OR (odds ratio) obtained from the logistic regression analysis using education level, employment, marital status, primi, informed decision on family size and having a female child as the independent variables; cases (unsafe abortion) and controls (unintended term pregnancy) as dependent variable

^c^Not included in the logistic regression model, as the variable either refers to only a sub-set of the sample or has smaller numbers (<10) in one category

^d^Calculated, for the employed

**Table 2:** Risk factors of unsafe abortion in relation to their family formation characteristics among women with unintended pregnancies

Family formation characteristics	Cases		Controls		Crude OR (95 % CI)	Adjusted OR (95 % CI)^b^
	*N* = 171^a^		*N* = 600^a^			
	No.	%	No.	%		
Current marital status						
Single/divorce/separate/widow	31	18.1 %	10	1.7 %	12.9 (6.3–27)	9.3 (4.0–21.6)
Married (Reference)	140	81.9 %	589	98.3 %	1.00	1.00
Fertility behaviour						
No decision on family size	89	52.0 %	173	28.8 %	2.5 (1.9–3.8)	2.2 (1.4–3.5)
Decision made:						
Family completed	41	24.0 %	230	38.3 %	0.9 (0.5–1.4)	1.0 (0.6–1.6)
Family not completed (Reference)	41	24.0 %	197	32.8 %	1.00	1.00
Ever used contraceptives						
No	37	21.6 %	96	16.0 %	1.5 (0.9–2.2)	-
Yes (Reference)	134	78.4 %	504	84.0 %	1.00	
Gravida						
Primigravid	36	21.1 %	83	13.8 %	1.7 (1.1–2.6)	2.2 (1.2–4.2)
Non-primigravid (Reference)	135	78.9 %	517	86.2 %	1.00	1.00
At least one male child						
No	65	38.0 %	221	36.8 %	1.0 (0.7–1.5)	-
Yes (Reference)	106	62.0 %	379	63.2 %	1.00	
At least one female child						
No	90	52.6 %	210	35.0 %	2.1 (1.5–2.9)	2.2 (1.4–3.4)
Yes (Reference)	81	47.4 %	390	65.0 %	1.00	1.00
Having disabled children						
Yes	6	3.5 %	8	1.3 %	2.9 (1.0–8.6)	-
None (Reference)	165	96.5 %	592	98.7 %	1.00	
In years	Mean	SD	Mean	SD	Significance	
Age at last pregnancy	30.6	6.6	30.5	6.3	*p* = 0.2	-
Marriage - P1 interval^c^	1.1	0.7	1.6	2.1	*p* = 0.3	-
Average birth interval^d^	3.4	1.6	2.9	1.2	*p* = 0.000	
Last birth interval^d^	5.7	4.1	4.8	3.3	*p* = 0.01	

^a^In some variables, row values do not add up to the total cases and controls due to missing data

^b^Adjusted OR (odds ratio) obtained from the logistic regression analysis using education level, employment, marital status, being primigravid, informed decision on family size and having a female child as the independent variables; cases (unsafe abortion) and controls (unintended term pregnancy) as dependent variable

^c^Only primigravid women included

^d^Only non-primigravid women included; average birth interval calculated since first pregnancy

With regards to socio-economic status, the risk of unsafe abortion was significantly higher among women who were less-educated (adjusted-OR = 1.5; 95 % CI = 1.1–2.4) or employed in unskilled/less-skilled occupations at the time (adjusted-OR = 2.3; 95 % CI = 1.4–3.6). Compared to the cases, their partners were better-educated, more employed and in skilled occupations. However, none of the characteristics of partners imparted any risk for women to undergo abortion, unless the partners were unemployed (unadjusted-OR = 6.3; 95 % CI = 1.5–26.3). This relationship too was not significant when adjusted for confounders.

A significant risk of unsafe abortion was seen among women in relation to some aspects of their family formation. The characteristics associated with abortion included being unmarried (adjusted-OR = 9.3; 95 % CI = 4.0–21.6), becoming pregnant for the first time (adjusted-OR = 2.2; 95 % CI = 1.2–4.2), not having made an informed decision on family size (adjusted-OR = 2.2; 95 % CI = 1.4–3.5) and not having at least one female child (adjusted-OR = 2.2; 95 % CI = 1.4–3.4) at the time of their unintended pregnancy.

**Fig. 1:**
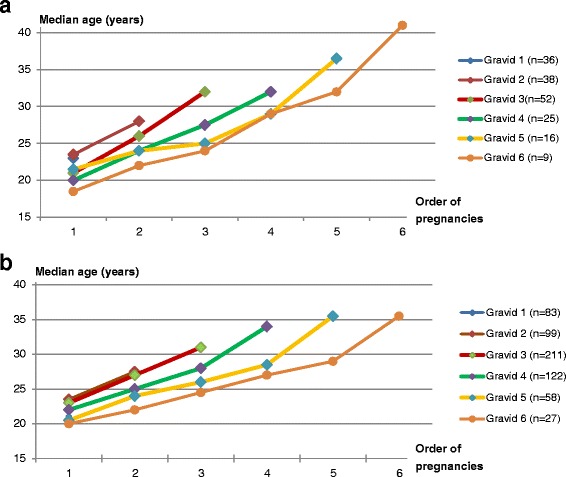
**a** Birth intervals of non-primigravid women following unsafe abortion (cases). **b** Birth intervals of non-primigravid women following unsafe abortion (controls)

**Fig. 2:**
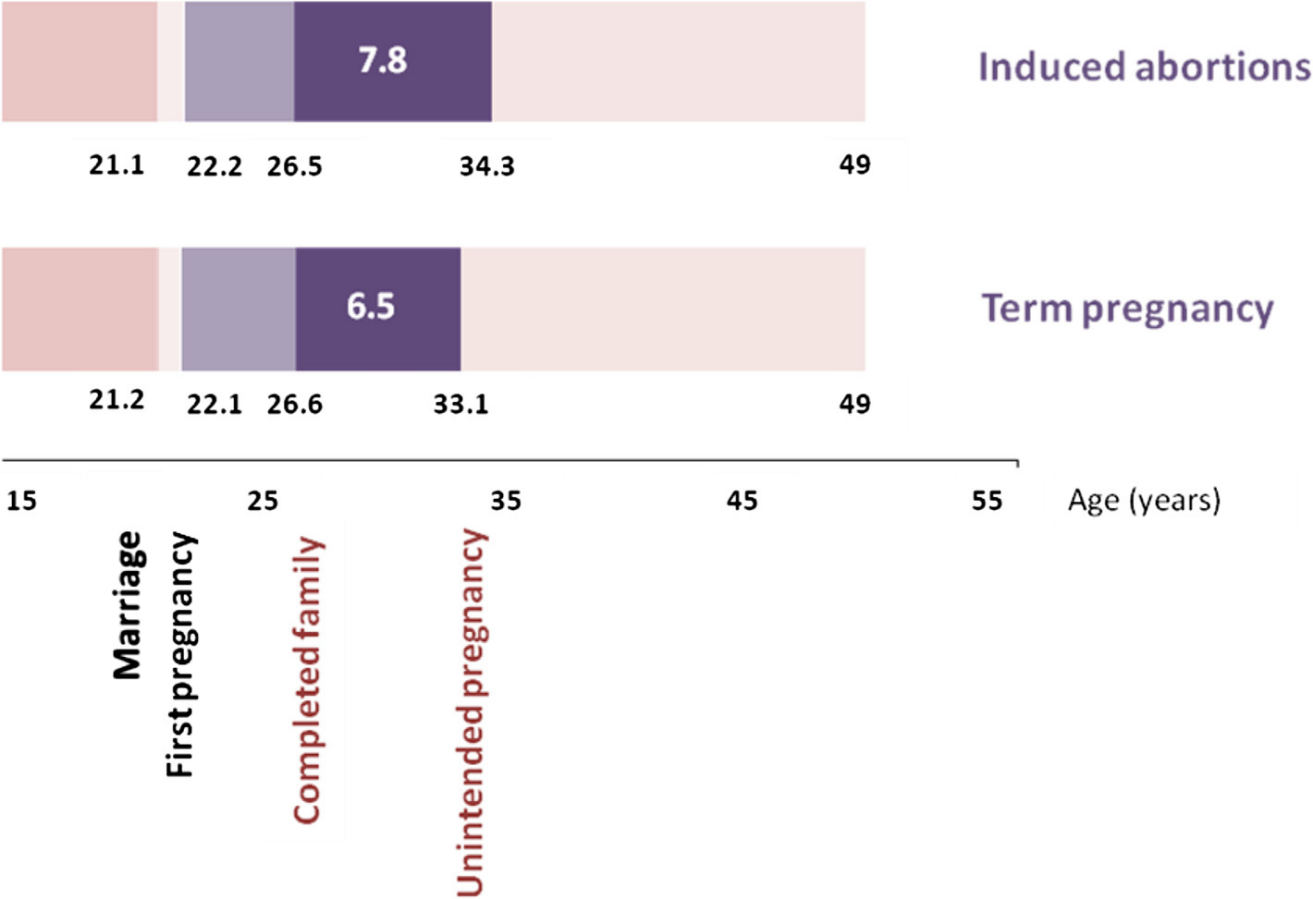
Events during the reproductive period of women who have their families completed

With regards to the risk of abortion associated with the timing of pregnancies, the average birth interval (Exp. ß = 0.7; 95 % CI: 0.6–0.8) was independently associated with abortion. In particular, non-primigravid women were significantly at risk of unsafe abortion, if they had longer average birth intervals (3.4 years in cases versus 2.9 years in controls) or longer last birth intervals (5.7 years in cases versus 4.8 years in controls). In further analysis, birth intervals of cases were all markedly longer (Fig. 1a) than the intervals of the controls (Fig. 1b), even when plotted against the order of pregnancies in each group of gravida (1–6). Cases were as fast as the controls in their family completion (4.3 versus 4.5 years, p = 0.4). No significant risk was noted in relation to age at which the women had their unintended pregnancy, first sexual encounter and first pregnancy (*p* > 0.05), as shown in Fig. 2.

## Discussion

Our study highlights that low socio-economic status, characterised by poor education and being employed in unskilled/less-skilled occupations is an independent risk factor for unsafe abortion. However, it is not the most influencing factor for unsafe abortions leading to complications, but many other aspects in relation to their family formation characteristics that are not confounded by their low socio-economic status, but act as independent risk factors for unsafe abortion. These factors include being unmarried, pregnant for the first time, not having decided on the desired family size, absence of a girl child and longer average birth intervals.

In comparison, in high-income countries where abortion is legalised, unsafe abortion is a risk among single, primigravid women of younger age [24, 25], while the low- and middle-income countries provide less-consistent evidence on these risks [3, 14]. Based on studies that have used comparative groups, the determinants of unsafe abortion identified in the low- and middle-income region are: younger [16] or older [17, 26] age at the time of abortion, younger age at first sexual act [17], unmarried [16], fewer [16] or many [17] living children, previous abortions [16], >1 partner [17], poverty [12], poor [12] or better [26] education, and use of contraception [26]. In the context of wide variation seen in these countries in relation to the data sources, the legal status of abortion and accessibility to safe abortion services, these findings need cautious application. In addition, the risks are not reported with adjustments made for their confounders.

In Sri Lanka, the determinants of induced abortion identified in one of the recent studies were older age, being married, young age of last child, having grown-up children, completed family and socio-economic constraints [27]. Our findings were in contrast to these. This was a community-based study on women who claimed to have undergone unsafe abortions (defined by any pregnancy terminated by an unskilled person or by a qualified doctor at a private place during last 18 months). Inferences drawn from this study are therefore less applicable for unsafe abortions, since the majority of women in this study would have undergone safely induced abortions or received relatively safe abortifacients from unskilled abortionists [28, 29]. Therefore, our study provides new knowledge on risk factors of unsafe abortion specifically among women with post-abortion complications.

Previous research has shown that low socio-economic status is the main risk factor for resorting to unsafe abortions [11–14]. This may be owing to the difficulty in affording more costly yet safer abortion services in countries where abortion is illegal. In concurrence, our study highlights the risk of abortion associated with poor economic status of women, characterised by their poor education (adjusted-OR = 1.5; 95 % CI = 1.1–2.4) and low-income occupations (adjusted-OR = 2.3; 95 % CI = 1.4–3.6). Difficulty in raising a child as well as fear of losing employment that had minimum job security could have been the predisposing factors associated with low socio-economic status for abortion. Previous studies have shown that less-urban districts having marginalised populations report higher proportions of unsafe abortion, reflecting the unavailability of safe abortion services [27]. Such studies conducted in hospitals or clinics providing free health care are known to represent patients predominantly of low socio-economic class, which could over-estimate the prevalence of low socio-economic status among abortion [14, 20, 30, 31]. We minimised this type of selection bias in our study, by comparing the cases with a comparable group of controls recruited from the same hospitals using similar selection criteria, when assessing the risk factors of unsafe abortion.

Unmarried status may prompt a woman to abort owing to difficulties in raising a child as a single mother. In contrast, we have shown in our study that this vulnerability of unmarried women for abortion was independent of their social status (adjusted-OR = 9.3; 95 % CI = 4.0–21.6). This reflects the social-stigma associated with ‘unwed mothers’ in Sri Lanka. Co-habitation outside marriage is not an accepted norm in the country [5]. In the current national family health programme, only the women in customary/legal marriage are entitled to domiciliary or clinic care by the public health midwife, unless they are pregnant or already having a child [5]. This excludes women not living together with their partners who have difficulty in accessing contraception services for preventing unintended pregnancies. Findings reiterate the importance of extending such services also to the unmarried women without causing any stigma.

Primigravid women in our study imparted a two-fold risk for unsafe abortions (adjusted-OR = 2.2; 95 % CI = 1.2–4.2), which indicated women’s desire for delaying their first childbearing. However, this finding is not in concurrence with the usual preference of Sri Lankan women, which is to bear the first child early, as the preference is shown to fall drastically from 77 % after marriage to 26.1 % after the first child [7]. It should be noted that this fact is relevant only for married women and that the behaviour of unmarried women following an unplanned pregnancy may not be the same. According to our study, unmarried status was also an independent risk factor for unsafe abortions, thus having some bearing on their desire for delaying their first pregnancy. On the other hand, the risk of abortion associated with being primigravid was independent of their poor socio-economic status, implying factors other than poor economic status for delay. Past decades have seen women changing attitudes on ideal family size [32] and becoming independent as much as their partners towards contributing actively to the family earnings and social commitments. This strife may be a likely reason for the risk of unsafe abortions in primis.

Previous research suggests that poverty predisposes a woman to restrict her family size, by resorting to abortion when faced with an unintended pregnancy [11–14]. In contrast, our study provides different views on their desired family size. Most disturbingly, women at risk of unsafe abortion were seen to be indecisive of their desired family size (adjusted OR = 2.2; 95 % CI = 1.4–3.5). This highlights a deficiency in the family planning programme in Sri Lanka. Primary objective of the family planning policy is to facilitate families to make informed decisions about their desired family size and to control their fertility through contraceptives [5]. This highlights the need to access newly-married couples for pre-pregnancy counselling and contraception services. Although eligible couple registration is almost 100 % in Sri Lanka [5], motivating couples to access pre-pregnancy services has been challenging for the public-health-midwife. The services need to be re-designed to make it more attractive and tailor-made, especially for working couples.

Young age of the last child that reflects a short last birth interval is a well-known risk factor for abortion [15, 27]. In contrast, our findings did not demonstrate this relationship. Instead, women having generally longer birth intervals, including the last one were at increased risk for abortion. This may imply the risk of abortion associated with the older age of the last child, which is likely to be a social stigma within the Sri Lankan society. Also, though family completion (26.5 %) has been a common risk for abortion worldwide [14], it was not so according to our study. In contrast, inability to make an informed decision on their desired family size has been a strong risk factor for unsafe abortions (adjusted OR = 2.2; 95 % CI = 1.4–3.5). These findings may be an indication of the unmet need for contraception among women who have no definitive plans on family formation. Despite a remarkable new acceptor rate of 90 % for temporary contraceptive methods and a contraceptive prevalence of 64.4 % [5] in Sri Lanka, unmet needs do exist particularly among women on temporary methods for a long period, that compel them to discontinue abruptly with no alternative protection. Over the past 15–20 years in Sri Lanka, age at marriage has been increasing along with fertility reductions observed in all age groups across all socio-economic strata, demonstrating women’s inclination towards limiting the number of children relatively early in marriage [7]. This further highlights an unmet need for protection from unplanned pregnancies until such women reach their menopause. Unless they are protected with long-term or permanent contraceptive methods, unsafe abortions would be a recurring problem in Sri Lanka.

### Strengths and limitations of the study

Obtaining a diagnosis of unsafe abortion is crucial in settings where reporting is deterred by legal, ethical and moral concerns. Particularly, population-based studies suffer from ‘misclassification’, as the diagnosis is made solely dependent on woman’s recall of past events [12, 33]. Hospital-based studies are also notorious for under-reporting [17, 34] but has its advantage of making a valid diagnosis based on clinical evidence following examination. In our study, we addressed under-reporting of unsafe abortions by restricting the cases to women in whom the diagnosis was confirmed by definitive clinical evidence and by obtaining data using medical graduates who were not involved in participants’ care but well-trained in conducting in-depth interviews. Furthermore, morbidity and mortality statistics owing to complications of unsafe abortion are reported predominantly from the state hospitals in Sri Lanka [5, 7]. Therefore, using state hospitals as the study setting provided a representative sample of women who were most at risk of morbidity consequences following an unsafe abortion. Though abortion services are not provided, state-owned hospitals provide post-abortion care, which are liberally accessed by women in the event of post-abortion complications, owing to free health services provided and the high health seeking behaviour among females in Sri Lanka. Only a small proportion would access non-state private health facilities for treatment of such complications following unsafe abortion.

## Conclusions

Poor education and employment in un/less-skilled occupations of women impart a significant risk for unsafe abortion among women faced with an unintended pregnancy. However, the risk of unsafe abortion associated with some aspects of family formation behaviour is seen to be independent of their low socio-economic status. In order to reduce this tendency, services should focus on reaching sexually-active women through pre-marital/pre-pregnancy counselling on the need for planning pregnancies and contraception before their first pregnancy, especially among the employed and poor women, and by inclusion of school-based education on reproductive issues and male participation targeting the adolescents in Sri Lanka.
